# RandoMice, a novel, user-friendly randomization tool in animal research

**DOI:** 10.1371/journal.pone.0237096

**Published:** 2020-08-05

**Authors:** Robin van Eenige, Peternella S. Verhave, Peter J. Koemans, Ivo A. C. W. Tiebosch, Patrick C. N. Rensen, Sander Kooijman

**Affiliations:** 1 Department of Medicine, Division of Endocrinology, Leiden University Medical Center, Leiden, the Netherlands; 2 Einthoven Laboratory for Experimental Vascular Medicine, Leiden University Medical Center, Leiden, The Netherlands; 3 Animal Welfare Body, Leiden University Medical Center, Leiden, The Netherlands; 4 Independent Consultant, Gouda, The Netherlands; 5 Animal Welfare Body Utrecht, Utrecht, The Netherlands; CIC bioGUNE, SPAIN

## Abstract

Careful design of experiments using living organisms (*e*.*g*. mice) is of critical importance from both an ethical and a scientific standpoint. Randomization should, whenever possible, be an integral part of such experimental design to reduce bias thereby increasing its reliability and reproducibility. To keep the sample size as low as possible, one might take randomization one step further by controlling for baseline variations in the dependent variable(s) and/or certain known covariates. To give an example, in animal experiments aimed to study atherosclerosis development, one would want to control for baseline characteristics such as plasma triglyceride and total cholesterol levels and body weight. This can be done by first defining blocks to create balance among groups in terms of group size and baseline characteristics, followed by random assignment of the blocks to the various control and intervention groups. In the current study we developed a novel, user-friendly tool that allows users to easily randomize animals into blocks and identify random block divisions that are well-balanced based on given baseline characteristics, making randomization time-efficient and easy-to-use. Here, we present the resulting software tool that we have named RandoMice.

## Introduction

Experimental research provides insight into cause-and-effect relationship by demonstrating what outcome occurs when a particular variable is manipulated. To be able to draw conclusions with high confidence, careful experimental design is critical to prevent systematic errors and randomization should, whenever possible, be part of it [1, 2]. Randomization involves the random assignment of experimental units–ranging from *in vitro* culture dishes to laboratory animals and human volunteers–to the various control and intervention groups. This ensures that any known and unknown covariate that might interfere with the outcome and therefore introduces bias, is randomly distributed over the experimental groups.

Power analysis allows us to determine the sample size required to detect an effect of a given size with a given degree of confidence. Nevertheless, for ethical and practical reasons we should always aim to keep the sample size as low as possible. Therefore, one might take the randomization of experimental units one step further and control for baseline variations in the dependent variable(s) and/or certain known covariates. This can be done by first defining blocks to create balance among groups in terms of group size and baseline characteristics, followed by the random assignment of blocks to the control and intervention groups.

The aim of the current study was to develop a user-friendly tool that allows users in animal research to identify well-balanced blocks based on the provided variables and covariates, and subsequently randomly allocate the blocks to experimental groups. Here, we present the resulting software tool that we have named RandoMice.

## Methods

The RandoMice software has been written in C# using the.NET Framework 4.7.2 and Microsoft Visual Studio version 16.4. The software has been developed as a single-user desktop application for machines running on Microsoft Windows. The source code of RandoMice has been published on GitHub [3] under the GNU General Public License version 3 (GPLv3), allowing for free usage, distribution and modification of the software under certain conditions [4].

### Main features of the RandoMice software

RandoMice has been designed to randomly divide experimental units into a given number of blocks of predefined sizes, a “block set”. The software will calculate a “ranking value” for each created block set which represents how well the blocks are balanced for the provided variable(s) and covariate(s). Block set creation stops when the predefined number of block sets are created or when all possible combinations are identified. When finished, RandoMice will show a list of the best-balanced block sets for the user to select the optimal block set for the experiment. To give a practical example, a researcher that aims to study atherosclerosis development in mice is now able to select a block set that is best balanced for the provided baseline characteristics, such as, but not restricted to, plasma triglyceride and total cholesterol levels and body weight. Subsequently, RandoMice can be instructed to randomly allocate the blocks to predefined experimental groups. The result sections will describe the work flow of the software and explain the main functionalities in detail.

### Statistical methods

Pearson product-moment correlation coefficients were determined to examine the relationship between measurements. Probability values less than 0.05 were considered statistically significant. All statistical analyses were performed using Prism GraphPad version 8.1.1 for Microsoft Windows.

## Results

**Fig 1:**
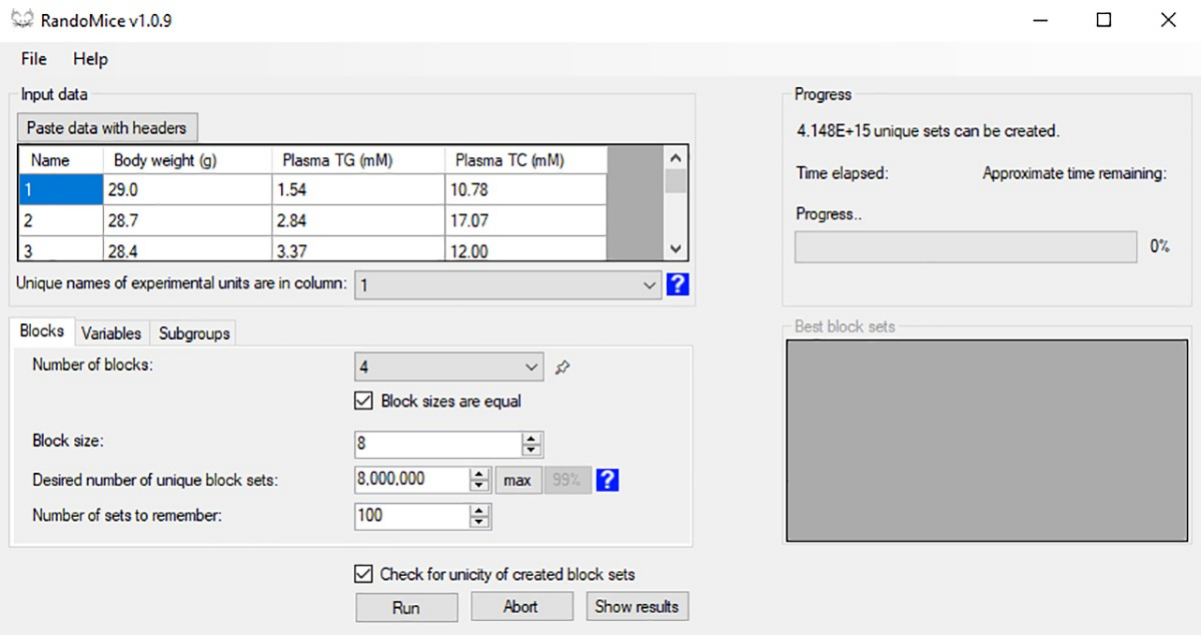
Screenshot of the RandoMice interface. Here, the user has imported data of experimental units into the software (top left section), and has defined the number of blocks and their sizes (bottom left section). With current settings, clicking the button “Run” will instruct the software to create 8 million unique block sets, each containing 4 blocks of n = 8 per block.

A screenshot of the RandoMice interface is shown in Fig 1. The full process is visualized in S1 Fig and referred to throughout the manuscript.

### Importing data and defining blocks

The RandoMice software can import data of experimental units from the clipboard, allowing for easy copy-pasting of such data from most spreadsheet editors. Each row should contain data of one experimental unit, with its unique name or number, variable(s), covariate(s) and optionally a physical marker (*e*.*g*. an ear mark or toe cut which is often used in experiments with rodents to identify the animals within a home cage; see below) in separate columns.

After importing data, the user should provide the desired number of blocks and block sizes (*e*.*g*. 2 blocks of n = 8 per block), see S1A Fig. Also, the user may set weights to each variable or covariate (S1B Fig, sub-panel a), which will reflect its relative importance (a larger weight corresponds with greater importance) when calculating the ranking value (see below).

### Defining subgroups based on physical markers

Physical markers, such as ear marks or toe cuts, are often used in experiments with rodents for genotyping purposes, and thereafter to identify the animals within a home cage. When animals are re-housed at the start of an experiment, re-marking may thus be required. To aid the user in this process, the user may provide such markers in one of the data columns, and indicate if division into subgroups based on the physical marker is required (S1B Fig, sub-panels b-c). This will instruct the software to show, for each block, the minimal number of physical markers that need to be modified in order to re-house the animals in the defined subgroups. The number of modifications is not part of the ranking value, but may be taken into account when selecting a block set (see below). If no markers are provided, the software will randomly divide the animals into the defined subgroups.

### Creating block sets

Systematically addressing all possible compositions of blocks will require substantial time and computing power as the number of experimental units within a block or the number of blocks increase; in most cases it will also not be necessary in terms of creating balance among groups (see below). Therefore, the RandoMice software is configured in such a way that it will randomly allocate the predefined number of experimental units to each block; a process that will be repeated as many times as instructed by the user.

**Fig 2:**
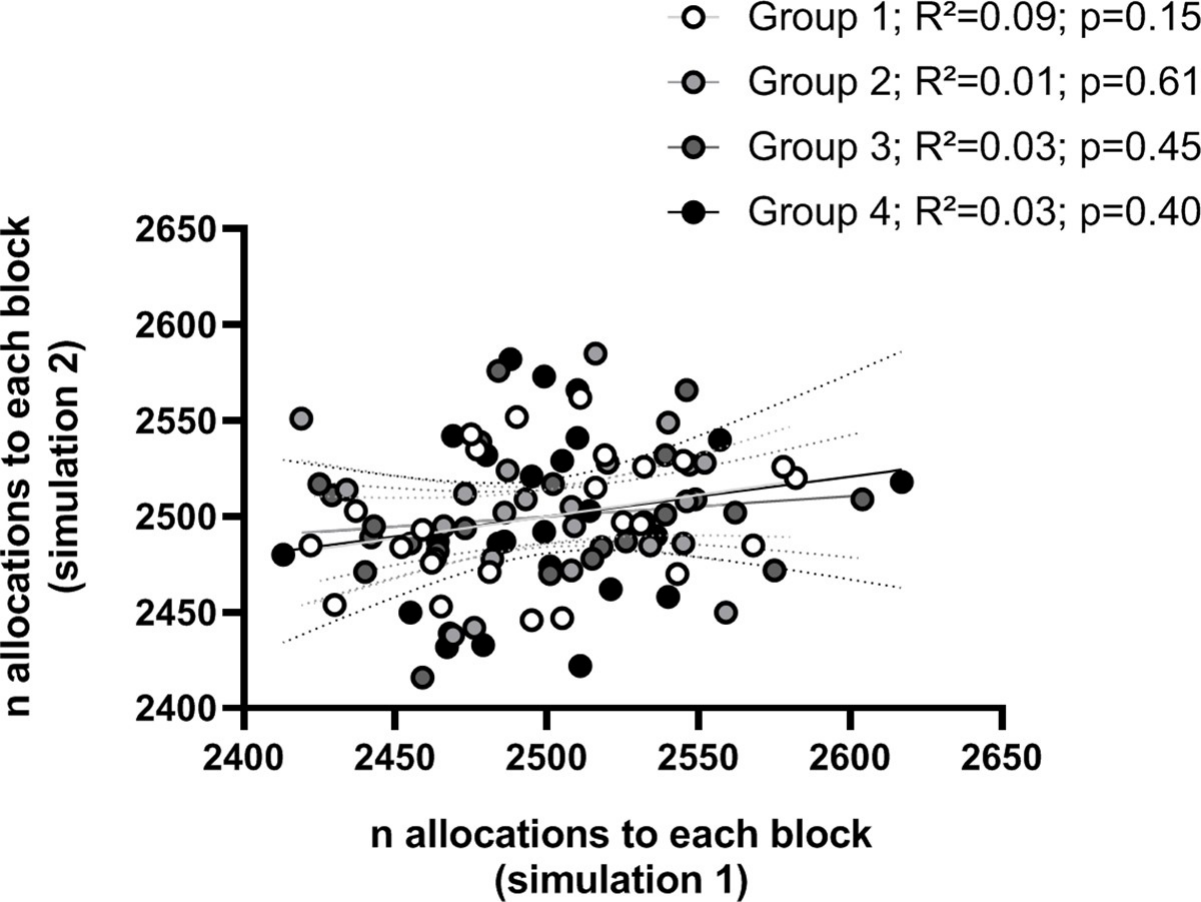
Validation of the randomness of the allocation process. Twenty-four dummy experimental units were repeatedly divided into four blocks of n = 8 per block to produce a total of 10,000 block sets (“simulation 1”) and another 10,000 block sets (“simulation 2”). For each block, the number of allocations of each experimental unit in simulation 1 was plotted against the number of allocations to that block in simulation 2. Pearson product-moment correlation coefficients were determined and linear regression lines were plotted with 95% confidence intervals (dotted lines). Data with p<0.05 were considered significant.

To determine if the allocation of experimental units to the blocks is indeed occurring as a random process, we divided 24 dummy experimental units into 4 blocks of n = 8 per block and instructed the software to create 10,000 block sets (“simulation 1”). This process was repeated to create another 10,000 block sets (“simulation 2”). In this set-up, each experimental unit should be allocated approximately 2,500 times to each of the 4 blocks. In addition if this process is random, there should be no correlation between the number of allocations of experimental units to each of the blocks in simulation 1 and simulation 2. As expected, the number of allocations to each block was 2,500 ± 34–50, and no significant correlation between the results of the two simulations was found (R^2^ = 0.01–0.09; p = 0.15–0.61), suggesting that there is no non-random bias in the block set creation, see Fig 2.

### Check for unicity of block set

Unless the user deselected the option to check whether a created block set is unique (S1B Fig), RandoMice will calculate a unique hash for each block set that is saved in memory and is used to prevent that identical block sets are scored more than once.

### Ranking block sets

The next step for the software is to calculate a ranking value for each block set, which reflects how well the blocks are balanced for the provided variable(s) and covariate(s). To determine the ranking value of a block set, differences between blocks in terms of averages and dispersion are considered, as well as the weights that the user has previously set. We have defined the ranking value as follows, see also Eq 1. First, for each variable and/or covariate (with $n\in N_{1}$) the average value and coefficient of variation (CV) per block and the standard deviation (SD) for the entire block set is calculated. Then, the maximal difference between the averages is determined. This value is normalized by expressing it as a proportion of the SD of the entire block set. To this, the maximal difference between the CVs is added to account for differences in dispersion between the blocks. The resulting value is multiplied by the weight that the user has previously set. Finally, the ranking value of a block set is calculated as the sum of the resulting values of each variable and covariate. By default, RandoMice will only remember details of the 100 best-balanced block sets, that is, the block sets with the lowest ranking values.

$${RankingValue}_{BlockSet}=\sum_{i=1}^{n}\left((\frac{{\Delta Mean}_{Max,i}}{{SD}_{BlockSet,i}}+{\Delta CV}_{Max,i})*{Weight}_{i}\right)$$ (1)

As an example, we have divided 16 dummy experimental units with three covariates and a physical marker (see S1 Table for details) into two blocks of n = 8. This means that a total of 6,435 unique block sets may be created. RandoMice was set to identify 99% of all possible combinations and to calculate the ranking value with equal weights for each covariate. This is the setup as shown in S1 Fig. In the block set with the lowest ranking value the mean and SD for each covariate was comparable between the two groups (4.38±1.71 *vs*. 4.48±1.72 for covariate 1, 14.58±2.31 *vs*. 14.56±1.87 for covariate 2, 1.19±0.33 *vs*. 1.20±0.36 for covariate 3), and to divide the blocks into two subgroups no physical markers needed to be modified (S1D and S1E Fig).

**Fig 3:**
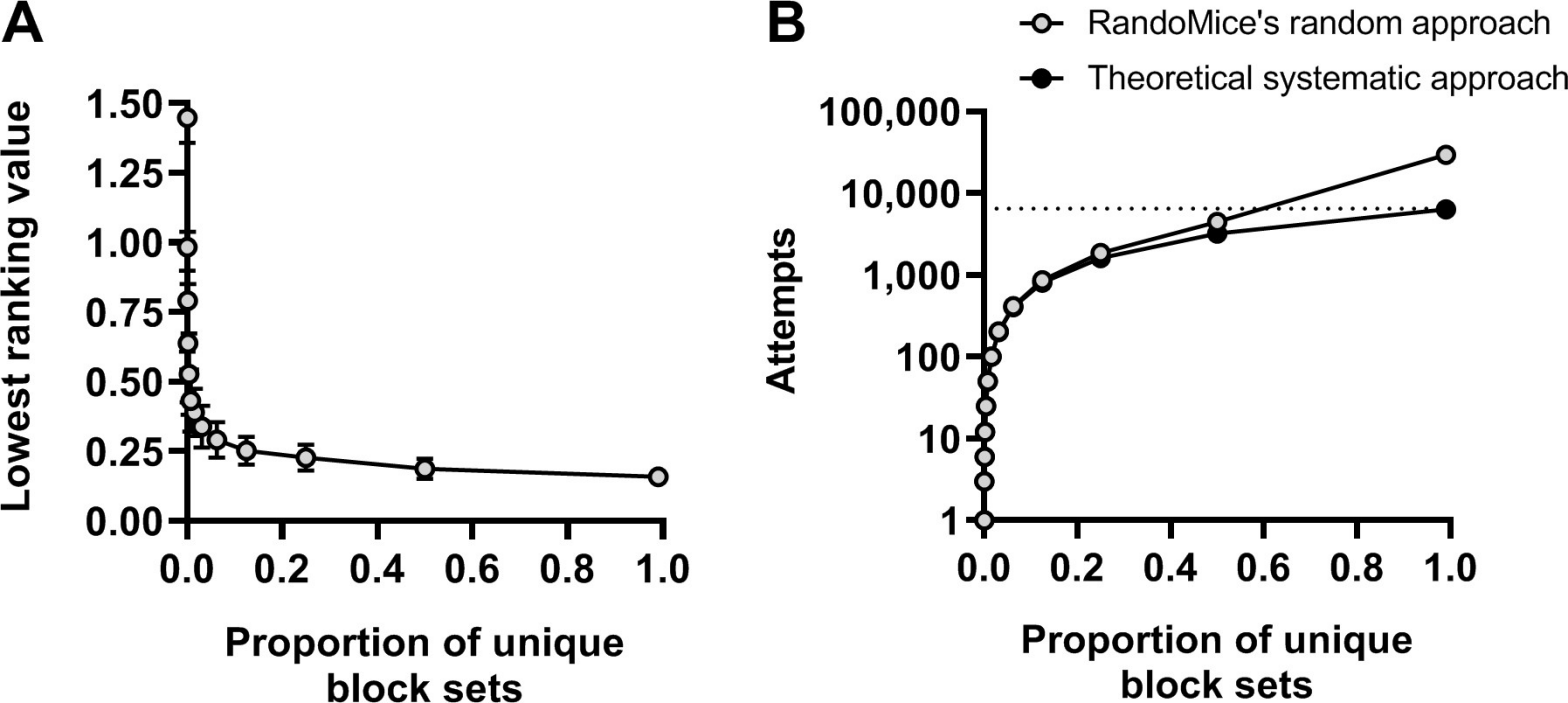
Assessment of the ranking value’s behavior when only a selection of all available block sets is identified. Sixteen experimental units with three covariates, as depicted in S1 Table, were repeatedly divided into two blocks of n = 8 per block, in which various proportions (see S2 Table) of all available unique block sets were created. We plotted **A)** the lowest identified ranking value and **B)** the total number of block sets created against the proportion of total unique block sets evaluated. Data are presented as mean ± SD, n = 256 per data point.

As mentioned before, increasing block sizes and/or the number of blocks may significantly increase the number of unique block sets and thus the required computational power and time to create all unique block sets. To assess the behavior of the ranking value when only a selection of all available block sets is identified, we repeatedly divided the 16 dummy experimental units with three covariates and a physical marker (see S1 Table for details) into two blocks of n = 8 per block and plotted the lowest identified ranking value against the proportion of unique block sets created, see Fig 3A. We also plotted the total number of attempts that were needed to identify a number of unique block sets and compared it to the theoretical number of attempts if we would have used a systematic approach to create block sets, see Fig 3B.

From these data we can conclude that increasing the time available to RandoMice yields block sets that are increasingly well-balanced for the provided covariates, but that on average creating 0.093% of all unique block sets is already sufficient to achieve approximately half (51% ± 19%) of maximal reduction in rank compared to fully random allocation, and creating 6.2% of all unique block sets is sufficient to achieve 90% ± 5% of maximal reduction. We can also conclude that RandoMice’s random approach to create block sets requires more attempts than a systematic approach. However, this only becomes apparent when creating >50% of all unique block sets, while at that point the chance that not any block set with a ranking value that is within the top 10 lowest ranking values was discovered is <0.1%. To put all of this in perspective, in this example, identifying 50% of all unique block sets only takes about a quarter of a second on any average computer.

### Selecting a block set and allocation to intervention groups

When the creation of block sets is finished, the RandoMice software will list the best-balanced block sets, displaying their ranking values and, if subgroups were defined, the total numbers of overlapping markers (S1D Fig). More detailed information such as block composition and averages may also be viewed (S1E Fig). At this point, the user should select his/her favorite block set and instruct the software to randomly allocate the blocks to the predefined interventions groups.

## Discussion

Carefully designing experiments using living organisms, either animals or humans, is of critical importance from both an ethical and a scientific standpoint. Randomization is an integral part of experimental design to reduce bias and thereby increase reliability and reproducibility [1, 2, 5, 6]. Although the need is acknowledged, the practical execution is often a bottleneck or a real challenge due to lack of easy-to-use tools. Some commercially available software packages such as IBM SPSS Statistics or Prism GraphPad offer tools that allow for randomizing experimental units into groups. However, balancing more than one variable or covariate over experimental groups remains challenging. In the current study, we designed a specialized, user-friendly tool that allows users in animal research to identify well-balanced blocks based on provided variables and covariates, and allows users to subsequently randomly allocate the blocks to experimental groups.

Besides improving study design, RandoMice can help users refining their animal experiments by displaying the number of overlapping physical markers in predefined subgroups. Creating physical markers, such as ear clips or toe cuts in mouse studies, temporarily cause animals discomfort and any additional modification should therefore be avoided. By providing the number of overlapping markers, the user can take this number into consideration when choosing from the top best-balanced block sets. Of course, in general one should always carefully consider the consequences of randomization if subsequent re-housing is required, as re-housing in itself may induce stress.

We compared RandoMice’s random approach to generate block sets to a hypothetical systematic approach. We showed that creating only a small proportion of all unique block sets is sufficient to identify a well-balanced block set. Although a systematic approach may be more efficient when the goal would be to identify all unique block sets, in many cases this will be practically impossible. For example, 4 blocks of n = 16 from 64 experimental units already yields 1.8·10^36^ unique possibilities. In such cases, systematically addressing only a proportion of all unique block sets would not be recommended as it will introduce a systematic bias. Another factor to consider is that with larger block sizes, the probability of randomly creating an imbalanced block set decreases thus reducing the need of identifying all unique block sets. We therefore conclude that random allocation of experimental units to blocks, as used in RandoMice, is the most efficient and least error prone approach for most practical situations.

RandoMice’s ranking method in its current form assumes that variables and covariates of experimental units are normally distributed continuous data. The user should be aware of this limitation and may need to test for normality and transform data before importing in RandoMice. Data that is not normally distributed, binominal values and other non-continuous data will not be treated differently from continuous data, which may cause imbalance among blocks.

To summarize, the RandoMice software is published on GitHub [3] under the GNU General Public License version 3 (GPLv3) [4], allowing anyone to freely use the program in the hope that it will be useful as a tool to refine experiments with living organisms more easily and thereby reduce the number of animals needed.

## Supporting information

S1 Table Input data for RandoMice to generate the results presented in Fig 3, S2 Table and S1 Fig. (PDF)

S2 Table Sixteen experimental units with three covariates and a physical marker, as depicted in S1 Table, were repeatedly divided into two blocks of n = 8 per block, in which various proportions of all available unique block sets were created. We determined the lowest identified ranking value and the total number of block sets created as a proportion of total unique block sets evaluated. The data presented here were used to create Fig 3A and 3B.
(PDF)

S1 Fig Screenshot of the RandoMice software. The screenshot is divided into five panels. **(A)** Here, the user has imported data of sixteen experimental units with three covariates and a physical marker, as depicted in S1 Table, into the software and **(B)** has instructed the software to create 99% of all theoretically available unique block sets, each containing two blocks of n = 8 experimental units per block. **(B, sub-panel a)** The number of decimal places of each covariate was two; the weight of each covariate was kept at one. **(B, sub-panels b-c)** The software was instructed to divide each block into two subgroups of n = 4 experimental units per subgroup, based on the physical markers. **(C)** While creating block sets, progress is displayed, and when finished running, **(D)** the software lists the 100 best-balanced block sets together with the number of overlapping physical markers and the ranking value. For each block within the currently selected block set, **(E)** the composition, the subgroup composition, as well as the mean and standard deviation of each covariate is displayed. At this point, the user should select his/her favorite block set and instruct the software to randomly assign the blocks to intervention groups.
(TIF)

## References

[1] van der Worp HB, Howells DW, Sena ES, Porritt MJ, Rewell S, O'Collins V, et al Can Animal Models of Disease Reliably Inform Human Studies? PLoS Medicine. 2010;7(3):e1000245 10.1371/journal.pmed.1000245 20361020 PMC2846855

[2] Smith AJ, Clutton RE, Lilley E, Hansen KEA, Brattelid T. PREPARE: guidelines for planning animal research and testing. Laboratory Animals. 2017;52(2):135–41. 10.1177/0023677217724823 28771074 PMC5862319

[3] RandoMice v1.0.9. GitHub. 9 April 2020 [Cited 22 April 2020]. Available from: https://github.com/Rve54/RandoMice/releases/.

[4] GNU General Public License version 3. GNU Operating System. 29 June 2007 [Cited 22 April 2020]. Available from: https://www.gnu.org/licenses/gpl-3.0.en.html.

[5] van Luijk J, Bakker B, Rovers MM, Ritskes-Hoitinga M, de Vries RBM, Leenaars M. Systematic reviews of animal studies; missing link in translational research? PLoS One. 2014;9(3):e89981–e. 10.1371/journal.pone.0089981 24670965 PMC3966727

[6] Hirst JA, Howick J, Aronson JK, Roberts N, Perera R, Koshiaris C, et al The Need for Randomization in Animal Trials: An Overview of Systematic Reviews. PLoS One. 2014;9(6):e98856 10.1371/journal.pone.0098856 24906117 PMC4048216

