# Supplementary material for: RandoMice, a novel, user-friendly randomization tool in animal research
Source: PLoS One. 2020 Aug 5;15(8):e0237096. doi: 10.1371/journal.pone.0237096 (PMC7406044; doi:10.1371/journal.pone.0237096)
Supplement: S1 Table — (PDF) [file pone.0237096.s001.pdf]

**Name of experimental unit**	**Covariate_1**	**Covariate_2**	**Covariate_3**	**Marker**
**1**	6.87	17.91	0.91	RRL
**2**	3.92	15.96	1.09	RL
**3**	5.59	15.07	1.24	RL
**4**	2.28	13.49	1.42	LL
**5**	2.10	12.30	1.36	L
**6**	5.24	9.90	0.70	RR
**7**	5.96	14.67	1.82	R
**8**	2.22	16.36	1.52	R
**9**	5.37	14.16	0.92	RLL
**10**	2.31	14.76	1.41	R
**11**	3.80	12.44	0.70	RRL
**12**	4.53	13.67	1.66	L
**13**	5.55	16.88	1.13	LL
**14**	5.55	13.75	0.80	RR
**15**	6.71	16.96	1.26	R
**16**	2.94	14.84	1.14	L
