# Supplementary material for: RandoMice, a novel, user-friendly randomization tool in animal research
Source: PLoS One. 2020 Aug 5;15(8):e0237096. doi: 10.1371/journal.pone.0237096 (PMC7406044; doi:10.1371/journal.pone.0237096)
Supplement: S2 Table — We determined the lowest identified ranking value and the total number of block sets created as a proportion of total unique block sets evaluated. The data presented here were used to create Fig 3A and 3B. (PDF) [file pone.0237096.s002.pdf]

**Proportion of unique block sets**	**Lowest ranking value**	**SD**	**n**	**Attempts (proportion of unique block sets)**	**SD**	**n**
**0.99**	0.16	0.0062	256	4.59	0.11	256
**0.50**	0.19	0.037	256	0.69	0.0070	256
**0.25**	0.23	0.047	256	0.29	0.0024	256
**0.12**	0.25	0.050	256	0.13	0.0012	256
**0.062**	0.29	0.064	256	0.064	0.00057	256
**0.031**	0.34	0.076	256	0.032	0.00030	256
**0.016**	0.39	0.085	256	0.016	0.00014	256
**0.0078**	0.43	0.11	256	0.0078	0.000072	256
**0.0039**	0.53	0.15	256	0.0039	0.000032	256
**0.0019**	0.64	0.21	256	0.0019	0.000010	256
**0.00093**	0.79	0.25	256	0.00093	0.000010	256
**0.00047**	0.98	0.38	256	0.00047	0	256
**0.00016**	1.45	0.55	256	0.00016	0	256
