# Supplementary figures and images for: RandoMice, a novel, user-friendly randomization tool in animal research
Source: PLoS One. 2020 Aug 5;15(8):e0237096. doi: 10.1371/journal.pone.0237096 (PMC7406044; doi:10.1371/journal.pone.0237096)

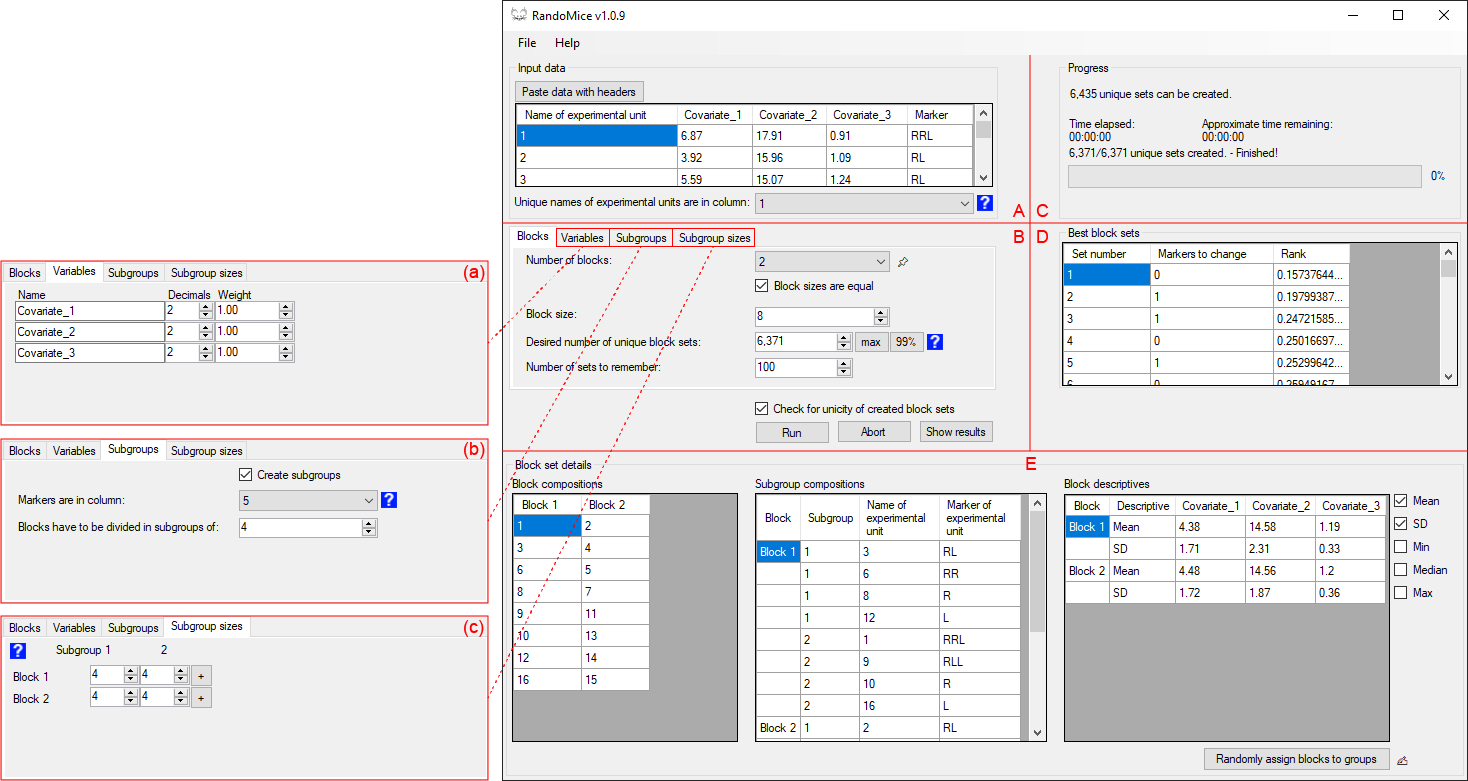

Supplement: S1 Fig — The screenshot is divided into five panels. (A) Here, the user has imported data of sixteen experimental units with three covariates and a physical marker, as depicted in S1 Table, into the software and (B) has instructed the software to create 99% of all theoretically available unique block sets, each containing two blocks of n = 8 experimental units per block. (B, sub-panel a) The number of decimal places of each covariate was two; the weight of each covariate was kept at one. (B, sub-panels b-c) The software was instructed to divide each block into two subgroups of n = 4 experimental units per subgroup, based on the physical markers. (C) While creating block sets, progress is displayed, and when finished running, (D) the software lists the 100 best-balanced block sets together with the number of overlapping physical markers and the ranking value. For each block within the currently selected block set, (E) the composition, the subgroup composition, as well as the mean and standard deviation of each covariate is displayed. At this point, the user should select his/her favorite block set and instruct the software to randomly assign the blocks to intervention groups. (TIF) [file pone.0237096.s003.tif]
